# The AP-1 clathrin adaptor has a conserved role in recycling resident proteins of the trans-Golgi network

**DOI:** 10.64898/2026.10.21.760840

**Published:** 2026-11-04

**Authors:** Fernando M. Valbuena, Maddison Lessard, Adam H. Krahn, Areti Pantazopoulou, Conor F. Lee-Smith, J. Christopher Fromme, Petia Adarska, Francesca Bottanelli, Benjamin S. Glick

## Abstract

Although the AP-1 clathrin adaptor has been studied for decades, its role in membrane trafficking has been ambiguous. Here, we systematically investigated the localization and function of mammalian AP-1. This adaptor associates with the trans-Golgi network (TGN) and with TGN-related recycling endosomes. Disruption of the μ1A subunit of AP-1 has only a mild effect on the Golgi export of secretory cargoes, but it causes depletion from the Golgi of multiple transmembrane TGN proteins. Live-cell imaging and a cell surface labeling assay indicate that when TGN proteins are unable to bind AP-1, they exit the Golgi to the plasma membrane. Our interpretation is that the vesicles generated with AP-1 recycle transmembrane TGN proteins, thereby keeping those proteins in the Golgi during cisternal maturation. The clathrin adaptor epsinR seems to function as a partner for AP-1 in recycling a subset of TGN proteins. These results closely align with studies of AP-1 function in yeast, suggesting that the conserved core role of AP-1 is the intra-Golgi recycling of TGN proteins.

